# Plastic and the Nest Entanglement of Urban and Agricultural Crows

**DOI:** 10.1371/journal.pone.0088006

**Published:** 2014-01-31

**Authors:** Andrea K. Townsend, Christopher M. Barker

**Affiliations:** 1 Department of Wildlife, Fish, and Conservation Biology, University of California Davis, Davis, California, United States of America; 2 Department of Pathology, Microbiology, and Immunology, School of Veterinary Medicine, University of California Davis, Davis, California, United States of America; CNRS, Université de Bourgogne, France

## Abstract

Much attention has been paid to the impacts of plastics and other debris on marine organisms, but the effects of plastic on terrestrial organisms have been largely ignored. Detrimental effects of terrestrial plastic could be most pronounced in intensively human-modified landscapes (e.g., urban and agricultural areas), which are a source of much anthropogenic debris. Here, we examine the occurrence, types, landscape associations, and consequences of anthropogenic nest material in the American crow (*Corvus brachyrhynchos*), a North American species that breeds in both urban and agricultural landscapes. We monitored 195 nestlings in 106 nests across an urban and agricultural gradient in the Sacramento Valley, California, USA. We found that 85.2% of crow nests contained anthropogenic material, and 11 of 195 nestlings (5.6%) were entangled in their nests. The length of the material was greater in nests in agricultural territories than in urban territories, and the odds of entanglement increased 7.55 times for each meter of anthropogenic material in the nest. Fledging success was significantly lower for entangled than for unentangled nestlings. In all environments, particularly urban, agricultural, and marine, careful disposal of potential hazards (string, packing and hay bale twine, balloon ribbon, wire, fishing line) could reduce the occurrence of entanglement of nestling birds.

## Introduction

The consequences of plastic accumulation for marine organisms are well-documented and have received wide attention [1]. Impacts of marine debris include transport of alien species [2] and pollutants to new locations [3], smothering of sea floor biota [4], and sorption and toxicity of contaminants [5]. Reports of entanglement and choking of marine wildlife are voluminous: more than 250 species are known to ingest or have been entangled in marine debris [6] including seabirds, turtles, fish, crustaceans, and cetaceans, sometimes with documented consequences for fitness [7] or population size [4].

In contrast, impacts of debris on terrestrial organisms are poorly documented and have been largely ignored, perhaps because terrestrial debris is less conspicuous [8] and more difficult to measure [9] than marine debris. As was initially the case with marine wildlife [7], entangled and choking terrestrial wildlife might be difficult to detect without deliberate effort, and opportunistic observations may go unreported [10]. Available data suggest that the impacts of debris on terrestrial organisms are similar to those on marine organisms, including sorption of chemicals [11] and mortality linked to ingestion [12]. Likewise, a number of studies report entanglement of terrestrial organisms, including snakes in beer can tabs [13], tortoises in balloon ribbon [10], and birds in anthropogenic nest material [14]–[16].

Potential effects of debris on terrestrial organisms could be most substantial in intensively human-modified landscapes (e.g., urban and agricultural areas). One of the few previous studies of nest entanglement in terrestrial birds, for example, reported that 4.6% of Osprey (*Pandion haliaetus* [15]) nestlings in an agricultural setting were entangled in anthropogenic nest material (twine). Likewise, urban settings are a primary source of trash entering marine [1] and terrestrial protected areas [17]. Historically, urban biotas have received very little attention [18], although interest has intensified in recent years in response to the rapid spread of urban landscapes across the earth's surface [19]. Nevertheless, the effects of plastic debris on urban organisms have been largely ignored. Impacts of debris could be magnified in urban adapted species, which are often characterized by their ability to utilize anthropogenic resources [20], [21]. A recent study of Chinese bulbuls *(Pycnonotus sinensis*), for example, showed that the proportion of anthropogenic material in nests increased with degree of urbanization [22]. The potential fitness consequences and adaptive significance of this adjustment, however, were not examined.

Herein, we examine the occurrence, types, landscape associations, and consequences of anthropogenic nest material in the American crow (*Corvus brachyrhynchos*; “crow” hereafter), a North American species that breeds in both urban and agricultural landscapes. This study is the first to explicitly document the links between terrestrial land cover, anthropogenic nest material, and nest entanglement in a terrestrial songbird.

## Methods

### Entanglement, fledging success, and nest analysis

**Figure 1:**
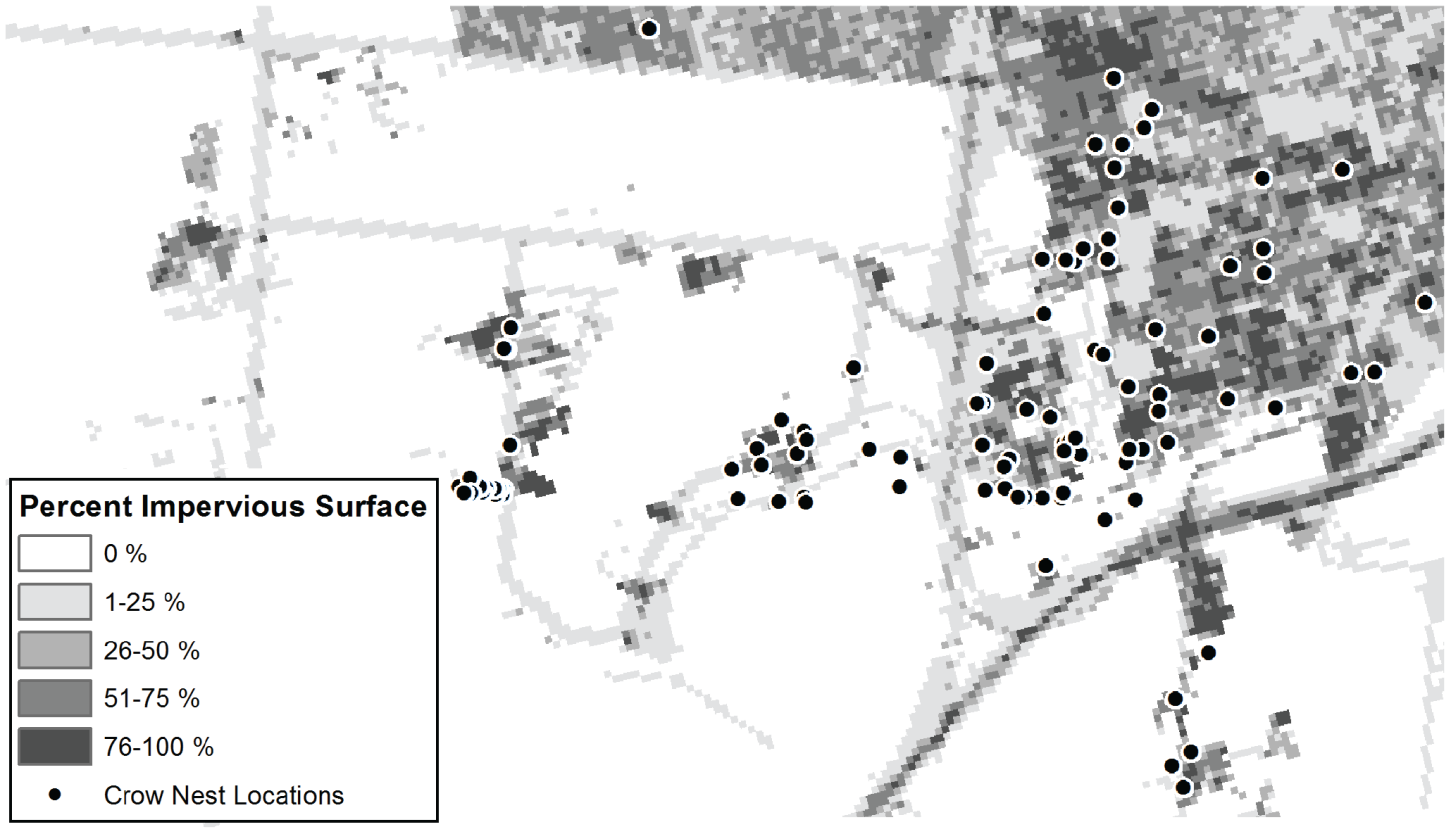
Map of study area in Davis, California, USA. All detected nests (*n* = 106) within this site were monitored for fledging success.

In 2012 and 2013, we monitored success of 106 crow nests across an urban to agricultural gradient in Davis, California. The study site spanned the urban campus of the University of California, Davis into the surrounding campus-owned agricultural areas (e.g., vineyards, pasture, and row crops; Fig. 1). Nests were situated on lateral tree branches (mean nest height ± SE: 9±0.5 m; *n* = 106 nests) and accessed by boom lift. Nestlings were checked for entanglement in the nest either once (approximately day 25 after hatching; *n* = 45 nests) or twice (<17 days after hatching and again on approximately day 25 after hatching; *n* = 39 nests). Entanglements were removed from live nestlings. Surviving nestlings were marked 25 days after hatching with a unique combination of color bands and a USGS band. After banding, nests were checked daily to monitor success or failure. The encounter rate of entangled nestlings represents a minimum entanglement rate, because dead, entangled nestlings could have been removed by parents or predators prior to nest checks.

A subset of 54 randomly selected nests was collected after the nestlings fledged or failed. We identified and measured the length (to the nearest 0.5 cm) of each piece of anthropogenic material longer than 5 cm from the lining of each nest. Corresponding nestling entanglement data were available from 32 of these nests. We had no corresponding entanglement data from 22 of these nests because the broods failed (i.e., they were depredated or failed from other causes, which could have included entanglement) prior to the first nestling check.

To assess how the probability of entanglement varied with amount of anthropogenic nest material, we analyzed entanglement of individual nestlings (0/1) as the response in a generalized linear mixed model (GLMM; binomial error, penalized quasilikelihood method), with total length of anthropogenic nest material in the nest as the predictor. We specified nest as a random effect to account for non-independence among nestlings within a nest. We included in this analysis the subset of nestlings for which we had corresponding nest material data (*n* = 64 nestlings from 32 nests).

### Landscape characterization

To characterize territories as primarily urban or agricultural, we created 1 ha buffers surrounding each nest site (Fig. 1). We estimated the average percent impervious surface within each buffer using the 2006 National Land Cover Database, which was the most recent version and representative of the study area [23]. We defined urban territories as those covered by an average of more than 50% impervious surface and agricultural territories as having 50% or less impervious surface. To assess how the amount of anthropogenic material varied with territory type, we examined (1) the total summed length of all anthropogenic nest material in each nest, and (2) the longest individual piece of anthropogenic nest material in each nest, in separate linear models with territory type (urban, agricultural) as the predictor. Length of anthropogenic material was square-root transformed to meet the assumption of normality. All statistical analyses were run in R v.3.0.1 [24], including the sp and raster packages for spatial analysis. All means and model parameter estimates are given ± SE.

### Ethics statements

This work was performed under protocols approved by the Institutional Animal Care and Use Committee of the University of California, Davis (Permit Number: 16897). All work was conducted on the private property of the University of California, Davis. No protected species were sampled.

## Results

**Figure 2:**
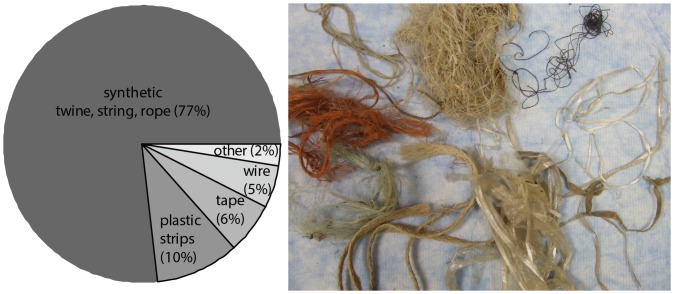
Percentage of each type of anthropogenic material in nests. Examples from nests are shown to right (*n* = 678 total items; 54 nests).

Anthropogenic material greater than 10 cm was detected in 85.2% (46/54) of dissected nests. Only three of 36 nests in primarily agricultural territories (8.3%) and five of 18 nests in urban territories (27.8%) contained no anthropogenic material. The mean total length of anthropogenic material in these 54 nests was 292.0±49.3 cm (range: 0–1858.3 cm). Material included synthetic string, twine, or rope; plastic tape; strips of plastic or cloth (including elastic, ribbon, gauze bandages, fabric straps, and unraveled woven sacks), fishing line, balloon string, unraveled nets, and wire mesh (Fig. 2). Amount of anthropogenic material in the nest varied with landscape: total length of anthropogenic material was significantly longer in nests in agricultural areas than in urban areas (mean total length agricultural = 363.70±68.04 cm; *n* = 36 nests; mean total length urban = 163.60±43.64 cm; *n* = 18 nests; β (urban) = −6.03±2.81; *t* (−2.15); *p* = 0.04), and the longest piece of anthropogenic material was significantly longer, on average, in agricultural nests than in urban nests (mean longest piece agricultural = 64.30±8.23 cm; mean longest piece urban = 38.04±8.32 cm; β (urban) = −2.18±1.02; *t* (−2.15); *p* = 0.04).

**Figure 3:**
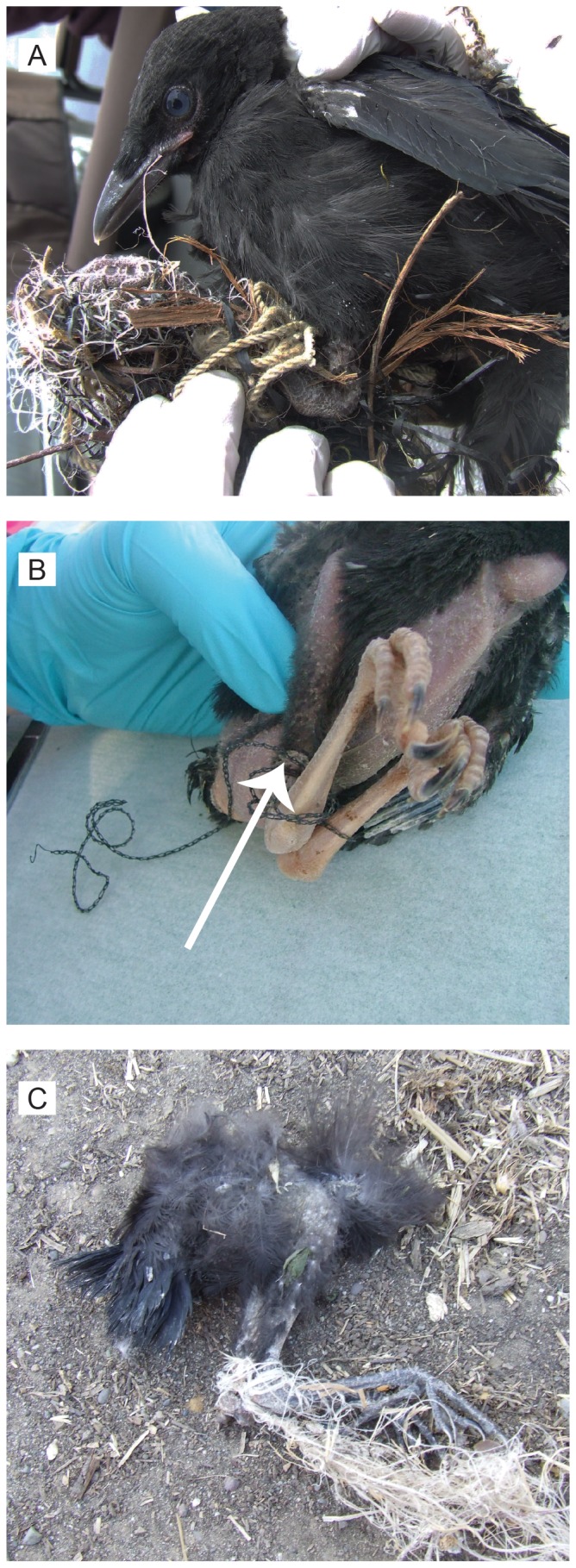
Nestling entanglement. (A) Nestling with tarsometarsus entangled in mass of synthetic string; (B) nestling with legs tied together with wire (arrow indicates strictures in the bone of the tibiotarsus); and (C) carcass of nestling with legs tied together by synthetic string.

Eleven of 195 nestlings (5.6%) were entangled (Fig. 3). The likelihood of entanglement increased with the total length of anthropogenic material in the nest (GLMM, 2.10±0.82 m; *t* (2.53); *p* = 0.017): the odds of entanglement increased 7.55 times for each meter of anthropogenic material in the nest. Nine nestlings were discovered entangled while still in the nest, and two were discovered dead below their nest tree. Entanglements were removed from all live nestlings, but in most cases, birds showed evidence of entanglement-related injury (e.g., strictures in the bone of the tibiotarsus (Fig. 3b); malformed toes). All nestlings that had been entangled (100%; 11/11 nestlings) failed to fledge, and the likelihood of fledging was significantly lower for birds that had been entangled than nestlings that had not been entangled (54.9%; 101/184 unentangled nestlings; χ^2^ (6.8) = 0.009; 2-sample test for equality of proportions with continuity correction).

## Discussion

In an early review of entanglement and ingestion of plastic debris by marine organisms, Laist (1987) listed three reasons why the significance of marine debris had been disregarded, all of which could apply to terrestrial organisms today: (1) the mechanics of entanglement were so straight-forward that they lacked “hidden mysteries;” (2) encounters between debris and wildlife could have been rare; and (3) the paucity of published reports appeared to confirm this overall rarity. Since that review, however, awareness and concern about the prevalence and problems associated with plastics in the marine environments have grown rapidly, and entire journal issues have been devoted to the topic [8]. Mechanical impacts of plastics on terrestrial organisms, however, are still largely disregarded.

We have shown that 85.2% of crow nests along an urban to agricultural gradient contained anthropogenic material, that the amount of material was higher in nests in agricultural areas, and that the likelihood of entanglement increased with length of anthropogenic material. All entangled nestlings (5.6% of the nestlings marked in this study) failed to fledge. Potential for entanglement and associated mortality could be widespread in birds in highly human-dominated (urban and intensive agricultural) landscapes. Anecdotal descriptions of anthropogenic nest material, including string, balloon ribbon, fishing line, plastic bags, paper, and dental floss, have been reported for many of the North American avian species defined as farmland species [25] (e.g., *Charadrius vociferous* [26], *Zenaida macroura* [16], *Cardinalis cardinalis* [27], *Sturnus vulgaris* [28], *Passer domesticus* [29]) *and/or urban exploiters and adapters* [20] (e.g., *Aeronautes saxatalis* [30], *Poecile rufescens* [31], *Psaltriparus minimus* [32], *Mimus polyglottos* [33], *Calypte anna* [34], *Haemorhous mexicanus* [35], *and Melozone crissalis* [36]). In some of these species, anthropogenic material has been anecdotally linked to entanglement or poor nest success [16], [32], [37].

Birds in urban and agricultural settings may use hazardous anthropogenic materials because they resemble their preferred, natural nest material (e.g., vines, grasses, strips of bark), analogous to marine turtles ingesting plastic bags because they resemble their jellyfish prey [38]. Some authors have suggested that highly modified environments, in general, could be ecological traps: animals are attracted to settle on the basis of historically adaptive cues, but cannot sustain a viable population because of low habitat quality [39], [40]. Entanglement in anthropogenic nest material can be added to the suite of documented stressors of urban and intensive agricultural landscapes, including toxins [41], novel predators [42], pesticide usage [43], tillage [44], roads [45], and disease [46].

In some situations, anthropogenic nest material could be a beneficial resource, enabling nest construction in places where natural materials are limited [47]. Anthropogenic nest material could have other benefits: for example, cigarette butts incorporated into nests reduced the ectoparasite load of some urban birds [48]. How these potential benefits balance the entanglement hazards of some anthropogenic nest materials is unknown.

We found that the amount of anthropogenic nest material was greater in the agricultural landscape than the urban landscape, likely due to the ready availability of twine and shade cloth wire in the agricultural settings. The majority of crow nests across this urban and agricultural gradient contained some anthropogenic material, however, and nestling entanglement occurred across this land use gradient. In all environments, therefore, particularly urban, agricultural, and marine, careful disposal potential hazards (string, packing and hay bale twine, balloon ribbon, wire, fishing line) could reduce the occurrence of nestling entanglement.
